# Climatic and endothermic thermal stress adaptation may be a major driver of emerging and emergent multidrug resistant fungi

**DOI:** 10.3389/fmicb.2025.1575755

**Published:** 2025-09-08

**Authors:** Chibuike Ibe, Carolina Henritta Pohl

**Affiliations:** ^1^Pathogenic Yeast Research Group, Department of Microbiology and Biochemistry, University of the Free State, Bloemfontein, South Africa; ^2^Department of Microbiology, Faculty of Biological Sciences, Abia State University, Uturu, Nigeria

**Keywords:** temperature increases, emerging and emergent fungi, AFR, virulence, stress

## Abstract

**Background:**

The impact of climate change and increasing global temperature is contributing to the emergence of unknown and the reemergence of known fungal pathogens.

**Main body:**

In the process of adapting to the increasing global temperatures, some fungi have evolved multidrug resistance traits thus narrowing the therapeutic options in our antifungal arsenal. Interestingly, all emerging fungal pathogens and known emergent fungi are multidrug and pandrug resistant, suggesting that the drug resistance traits observed are partly due to thermal stress-induced cross stress-responses. This paper argues that the acquired drug resistance traits may also be driving increased virulence and adaptation to infection-related conditions, resulting in the outbreak of community and hospital infections.

**Conclusion:**

Continued surveillance and more research are required to enhance our understanding of the impact of heat-induced evolution of antifungal resistance in this rapidly evolving area of research that may define a new era in medical mycology.

## Introduction

For the vast majority of fungi, the inability to grow at elevated temperatures limits their capacity to infect human. Fungal pathogens common to immunologically competent human were rather those associated with superficial mycoses—tinea, oral thrush and (vulvovaginal candidiasis) VVC—which are non-fatal and amenable to antifungal therapies (Seagle et al., 2021). However, invasive human mycoses became common in the past three decades because of diseases that weaken the immune system such as diabetes and advanced medical interventions (Casadevall, 2018). Examples include the use of indwelling devices, and cancer therapies that impair the body’s immunity.

Currently, global warming is giving rise to extensive variation in temperatures that can alter cellular metabolism and genetic makeup of an organism (Seidel et al., 2024). These temperatures are higher (by about 2°C) than in the past two centuries with significantly hotter days that can act as selection events for the survival of certain organisms, altering membrane fluidity, mobilize heat shock proteins (Hsps), and cell surface remodeling enzymes and induce rapid mutagenesis leading to thermotolerance, thus eliminate environmental temperature difference with endotherms (Gong et al., 2017; Heilmann et al., 2013; Huang et al., 2024). This has remained increasingly worrisome.

## Opinion

**Figure 1:**
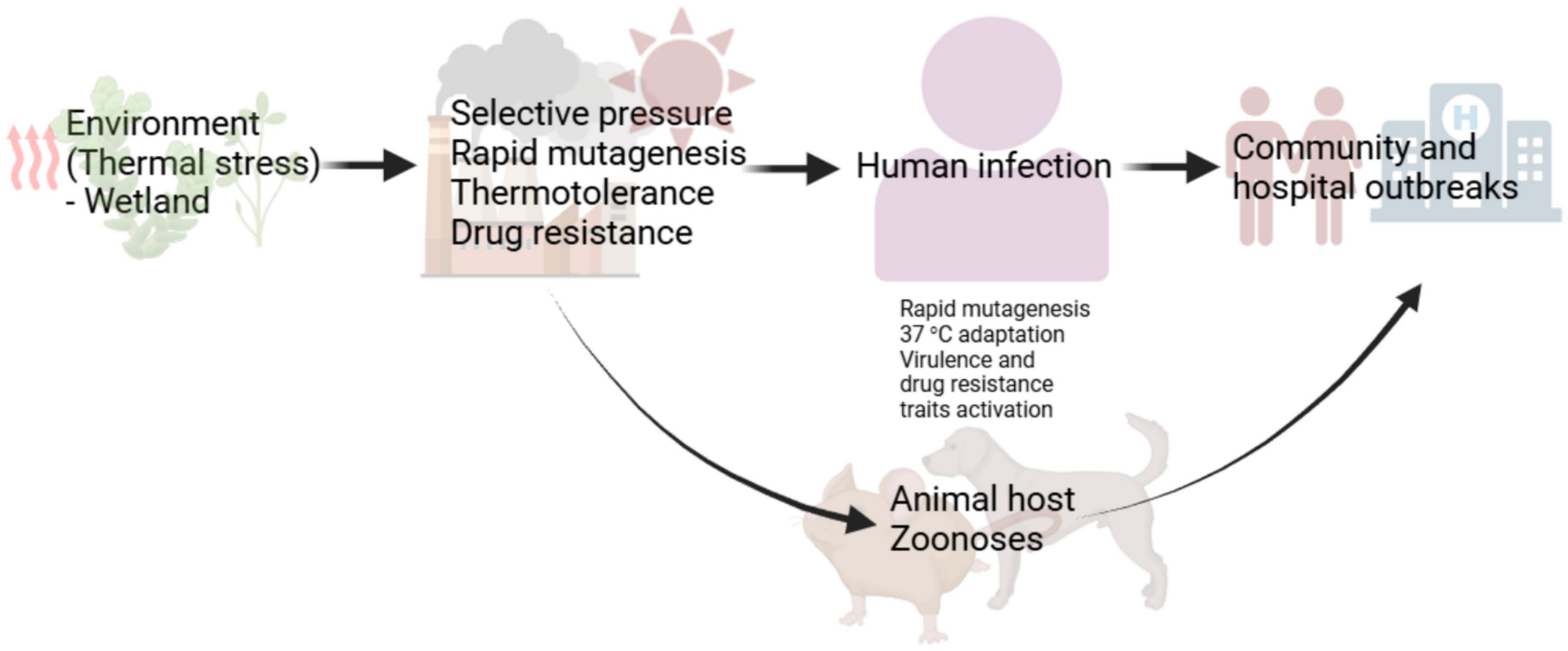
A proposed evolutionary path for emerging fungal pathogens. Emergent fungi become drug resistant before emerging in the community or causing hospital outbreaks. Thermal stress responses including the activation of heat shock proteins recruit transcription factors regulating salvage response pathways involved in drug tolerance and drug resistance. This may account for the emergence of drug resistant *Candidozyma auris*, *Rhodosporidiobulus fluvialis* and *Candida khanbhai*. The development of drug resistance may also follow the accumulation of mutation, due to thermal stress, that confer drug resistance through cross-stress advantages. Cross-stress responses can ultimately trigger the expression of latent or novel pathogenicity traits in the course of infection. Initial endothermic stress adaptation can induce hypervirulence and multidrug resistance traits development and expression.

The emergence of multidrug resistant *Candidozyma auris* has been a scientific mystery, especially, its independent occurrence in three continents where the fungus caused candidiasis (Lockhart et al., 2017). Though the mechanism(s) responsible for this is yet understood, many scholars have offered opinions on this phenomenon. While some of these opinions may differ, for example those providing evidence for (Casadevall et al., 2019) or against (Money, 2024) thermal stress as a contributing factor in the emergence and reemergence of fungal pathogens, they offer areas of potential continuing research. Data implicating thermal stress is compelling indicating that, climatic (Mora et al., 2022) or endothermic (Huang et al., 2024; Schwiesow et al., 2024; Casadevall, 2023) thermal stress adaptation is at least impacting the expression of virulence traits whether novel or latent in otherwise benign fungi. What we do not also understand yet is the extent to which this is occurring, and evidence suggests at a rather worrisome rate. An interesting argument is that emergent fungi acquire resistance traits in their evolutionary path which may entirely be environmentally induced (thermotolerance) before the opportunity to cause outbreaks through cross-stress advantages (Figure 1). For example, *C. auris* strains with fluconazole minimum inhibitory concentration (MIC) of >256 μg/mL, and amphotericin B MIC of 2–4 μg/mL have been isolated in the Andaman Island, India, in habitats without any human activities indicating its prior existence as a drug resistant environmental fungus (Arora et al., 2021). The survival of *C. auris* in wetland shows its tolerance to harsh environment (Casadevall et al., 2019). Microorganisms thermal stress adaptation alone may not fully explain the emergence of fungal pathogens. A combination of factors including the impact of thermal stress on host immune response which remains to be understood may also be at play. However, the ability to overcome the core human body temperature is important both in the development of drug resistance and expression of virulence traits (novel or latent) and in the ability to infect sufficiently immunocompromised hosts.

## Evidence supporting the independent emergence of drug resistance

Drug resistance can emerge in fungal pathogens without prior exposure to antifungal drugs or their analogues. Data has shown no correlation with azole agricultural use and the emergence of multidrug resistant *C. auris*; also, the emergence of azole-resistance in *Candida* spp. predates the emergence of the ‘auris’ species. Azole and polyene resistance in *C. haemolunii* var. *vulnera* a close relative of *C. auris*, without prior antifungal exposure, was shown to likely be due to Erg11 variant (Muro et al., 2012; Rodrigues et al., 2020). In addition, a survey of antimicrobial susceptibility profile of 1,698 reference fungal strains showed that fungal species recovered from fruits and insects already are resistant to major classes of antifungals with several showing pandrug resistance traits (Desnos-Ollivier et al., 2012). This is consistent with the data from the semi-arid Eastern Karoo climate region of South Africa, where *C. auris* isolated from the gut of *Locustana pardalina* (locust) were fluconazole resistant (32 μg/mL), survived growth at 50°C and tolerated 15% NaCl. The interesting thing here is that the locusts were collected from a remote rural area consisting of large private grazing with limited human activity suggesting that metabolic activities of thermotolerant insects can select for drug resistance traits in fungi.

## Evidence implicating thermal stress response genes and pathways in drug resistance and virulence

It has been argued that acquisition of drug resistance can occur independent of virulence, because they are controlled by different cell properties and simultaneous occurrence can lead to fitness defects (Vincent et al., 2013). Studies have shown that Hsps play a role in adaptation to heat stress. Heat stress responses by Hsps are generally regulated by an essential transcription factor, Hsf1. Hsf1 and transcription factors regulating cell surface stress-activated protein kinase pathways: Hog1, Mkc1 and Cek1, are thought to be Hsps (Hsp90) client proteins; with all potentially involved in thermotolerance in *Candida* spp. (Ibe and Munro, 2021). It can be argued that this heat shock-stress response regulatory circuit partly explains the emergence of a drug resistant fungal pathogen. The evolutionarily conserved Hog1 pathway linked to virulence in *Cryptococcus neoformans* has been shown to promote stress response and virulence in *C. auris* (Day et al., 2018), indicating a link between adaptation and virulence. In *Aspergillus fumigatus*, it has been hypothesised that antifungal resistance can drive virulence and adaptation to infection-related stress (Paul et al., 2019; Hagiwara et al., 2017). In addition, deletion of Hsps genes has shown altered traditional drug tolerance phenotype and attenuated virulence in *Candida* spp (Gong et al., 2017).

## Increasing temperature adaptation alters virulence and drug resistance traits

In a current study, Schwiesow et al. (2024) used Ftc239-1, an environmental isolate of *C. neoformans* strains with mild and severe growth defects at 30°C and 37°C, respectively, to understand the impact of adaptation to increasing temperatures on pathogenicity traits and drug resistance. To test tolerance, Ftc239-1 strains were evolved through 38 passages every 4–6 days with 0.5–1°C temperature increases to 30°C and 35°C. The evolved strains grew well at 35°C and even more substantially at 37°C than their parent strain and showed phenotypic heterogeneity. For example, the capsule sizes and capsule production were similar to the parent strain with one of the evolved strains producing substantially more titan cells (important in pathogenesis). The evolved strains had fitness advantages at high temperatures with genotypic heterogeneity. Interestingly, frameshift and point mutations were observed in *SSK2* and *PBS2*, respectively, which are key genes in Hog1 pathway highlighting the importance of the pathway in yeast stress response. Ftc239-1 had a fluconazole MIC of 8 μg/mL while the evolved strains MICs ranged from 3 to 16 μg/mL. MIC of the evolved strains showed that heat stress can have a complex effect on microorganisms however, MIC of 16 μg/mL indicates potential cross-stress advantages including reduced drug susceptibility. Strains with impaired ability to produce titan cells carried frameshift mutation in *LMP1*, the gene required for the production of titan cells, suggesting possible loss of function.

In *C. deneoformans*, data showed that the rate of mutation is higher at 37°C (with an 8–25 fold increase) than at 30°C. Interestingly, heat induced mobile genetic elements were shown to be the major drivers of microevolution and rapid adaptation to heat stress compared to point mutations and small insertions/deletions (Gusa et al., 2023). These genetic element mutations are the major source of stress induced genetic variation.

## Endothermic stress responses confer pandrug resistance and hypervirulence traits on yeast

The work of Casadevall in 2015 showed a negative correlation between thermal stress and antifungal resistance, however, a strong correlation existed between caspofungin resistance and basidiomycetes (Robert et al., 2015). Ten years after, data now show that ascomycetes can evolve drug resistance traits linked to thermal stress and may emerge as a human pathogen due to cross stress adaptation or the acquisition of new virulence traits or activation of latent ones (Money, 2024). For example, the first description of *C. auris* (amphotericin B: MIC, 0.5–32 mg/mL; fluconazole: MIC, 2–128 mg/mL) (Kim et al., 2009), was in an infection of human ear which is a cooler environment than the gut; current bloodstream infections may indicate successful adaptation to 37°C which induced virulence and mutagenesis that further conferred pandrug resistance traits on the fungus. This may partly explain the emergence of multidrug resistant *C. auris* and has recently been demonstrated in *Rhodosporidiobulus fluvialis*, a previously undescribed fungal pathogen (Huang et al., 2024), as well observed in *Candida khanbhai* a recently characterised member of the *C. haemulonii* species complex (De Jong et al., 2023).

Huang et al. (2024) showed that temperature-dependent mutagenesis can drive the development of virulence and intrinsic resistance to most antifungal therapies. Their research is the outcome of China’s surveillance to monitor the epidemiology and drug resistance patterns of bloodstream yeast pathogens through the CHIF-NET—China hospital invasive fungal surveillance net, programme. The programme identified infection with two clinical strains: NJ103 and TZ579, of *R. fluvialis*. *R. fluvialis* was first described in aquatic environment, vegetation and soil in different countries as a benign fungus before being isolated in two immunocompromised patients in China with 100% fatality. The authors used mouse-model of invasive fungal infection to show that only NJ103 and TZ579 of the genus *Rhodosporidiobulus* and the environmental yeast *R. nylandii* were able to adapt to 37°C and cause disease.

Intriguingly, in the course of mice infection with NJ103 and TZ579, part of their cell population was able to transition and grow in a more virulent psuedohyphae form which also evaded macrophage internalization than the yeast form causing mice mortality. Growth at 37°C induced the morphological transition (significant in pathogenesis); it also led to the accumulation of intracellular reactive oxygen species resulting in oxidative DNA damage and rapid acquisition of single nucleotide polymorphisms. NJ103 and TZ579 were resistant to fluconazole and echinocandins. However, the elevated mutation rates induced by growth at 37°C resulted in rapid emergence of resistance to amphotericin B and 5-fluorocytosine. While this is a worrisome outcome, making the clinical strains unusual but perhaps not unique, it showed that human body heat adaptation can drive rapid evolution of pandrug resistance in yeasts. The molecular basis of drug resistance in the pathogens is yet understood, however, whole genome sequencing showed duplicate copy of *ERG11* which has been implicated in fluconazole-resistance. Deletion of one *ERG11* copy did not affect the drug resistance phenotype. This indicates a complex drug resistance mechanism or the presence of a genetically unusual *ERG11* homologue in the fungus. Heterologously expressing the *ERG11* copy in *C. neoformans*, conferred fluconazole-resistance.

The *R. nylandii* strain was intrinsically resistant to caspofungin and fluconazole. However, adaptation to mammalian core body temperature induced amphotericin B resistance (Huang et al., 2024) leading to pandrug resistance. This further showed that temperature induced rapid mutagenesis can drive evolution of traits in fungi. *R. nylandii* is yet to cause any human disease but has the traits of known fungal pathogens.

Another example of emergent fungi developing drug resistance first in their evolutionary path is in the *C. haemulonii* species complex—all members are closely related to *C. auris* (all clustered in Metschnikowiaceae clade)—which has attracted attention because of their emerging multidrug resistance (Chen et al., 2022), high transmissibility in clinical settings and increasing worldwide emergence (Cao et al., 2023). Azole resistance in the species has been linked to *ERG11* substitutions and amplification suggesting the act of evolutionary pressure (Gade et al., 2020). A newly characterised member of the complex, *Candida khanbhai*, was recently identified from the nostrils of a Kuwaiti patient in 2019 and bloodstream of a Malaysian patient in 2014 as an emerging fungal pathogen (De Jong et al., 2023). This suggests that the fungus emergence and adaptation to 37°C may have begun by initially infecting cooler areas in humans just like *C. auris*. The impact of endothermic stress adaptation on drug resistance was also reflected in the strains drug susceptibility profile. Though both strains had elevated amphotericin B MIC values: 2–4μg/mL, the bloodstream strain showed a strong increase in MIC values: 4–16 μg/mL for flucytosine and moderately elevated MICs for anidulafungin (0.5–1 μg/mL) and micafungin (0.25–0.5 μg/mL) indicating that endothermic stress adaptation can further amplify drug resistance traits. Generally, both strains showed reduced susceptibility to multiple antifungals tested, however, an interesting observation by the authors, using *C. auris* breakpoint, was the clinical impact of trailing effect after 48 h which showed multidrug resistance patterns. This report is similar to that of *C. auris*, emerging from geographically distinct locations with multidrug resistance traits.

## Impact on antifungal therapy

Antifungal resistance (AFR) has become a serious global problem partly because antimicrobial resistance programmes have traditionally removed antifungals due to fungi being widely neglected as a public health threat. For example, the joint programme initiative on antimicrobial resistance (JPIAMR) consortium only included AFR in their agenda for strategic research and innovation on antimicrobial resistance in 2021 (Fisher et al., 2022). Despite the limited funding in antifungal research, our current antifungal arsenal, still has some very potent antifungals such as the echinocandin that very few yeasts including *Nakaseomyces glabratus* are intrinsically resistant to. However, emerging (e.g., *C. auris*) (Jacobs et al., 2022) and emergent (e.g., *R. fluvialis*) fungi (Huang et al., 2024) now show intrinsic resistance to echinocandin and to all the classes of available antifungals.

Acquisition and emergence of AFR which may originate from the environment (Huang et al., 2024) or the host (Shields et al., 2012) is fundamentally an evolutionary response to the selective pressures exerted by a drug. Mechanistically, AFR is usually acquired due to changes that directly or indirectly affect the drug–target interaction. These include genetic changes to the target, altering the drug concentration through drug efflux activity for azole (Robbins et al., 2017) or for flucytosine (Edlind and Katiyar, 2010) inhibition of prodrug activity, and overexpressing the amount of the target available. However, data have now shown the possibility, in the absence of any drug selective pressures, of temperature-induced rapid mutagenesis that results in multidrug and pandrug resistance in fungi (Huang et al., 2024). It is possible that the drug resistance traits in these fungi are products of unintended cross-stress advantages situated by thermotolerance and perhaps by other yet unknown factors. Data is yet to show if the accumulation of these kinds of mutations come at a fitness cost in the fungi in the future. However, it is currently negatively impacting therapeutic outcomes in fungal disease management (Takoutsing et al., 2023).

## Conclusion

Since most, if not all, emerging pathogenic yeasts emerged as multidrug resistant, it can be argued that their virulence traits may partly be due to cross-thermal-stress adaptation responses of evolved drug resistance traits which are further amplified upon infection. The direct impact of thermal stress adaptation on the evolution of fungal pathogens is yet understood though the risks remain high. Surveillance remains the cornerstone for controlling emerging fungal pathogens (Liao et al., 2024). in Asia, one of notable surveillance efforts is CHIF-NET through which 9,713 yeasts have been grown (8,829 *Candida* and 884 non-*Candida* yeast) with several rare or uncommon yeasts described, including *Trichosporon dohaense*, *Diutina catenulata* (*Candida catenulata*), *Yarrowia lipolytica* (*Candida lipolytica*) and *Kodamaea ohmeri*, and members of the Metschnikowiaceae clade (Bottery and Denning, 2024).

In Africa, several laboratories, are optimising protocols using culture-based and non-culture-based techniques in pilot studies for the surveillance of pathogenic yeasts in wastewater (Baker et al., 2025). Data so far suggests that the development of azole-resistance in yeasts is not necessarily through the selective pressure of environmental azoles but could be through adaptive responses that unknowingly confer drug resistance. Part of the surveillance efforts at the University of the Free State, South Africa, also include sampling freshwater plastisphere to increase our understanding of their yeast community which can be a vehicle for multidrug resistant pathogenic yeasts distribution and transmission (Baker et al., 2024). Further work is required in this rapidly evolving area of research.

## References

[ref1] Arora P.. (2021). Environmental isolation of candida auris from the coastal wetlands of Andaman islands, India. mBio 12, e03181–e03120. doi: 10.1128/mBio.03181-20 33727354 PMC8092279

[ref2] Baker T. Bester A. Sebolai O. Albertyn J. Pohl C. (2024). Biofilms on urban aquatic plastic pollution as a reservoir for pathogenic yeasts. J. Water Health 22, 1826–1842. doi: 10.2166/wh.2024.133

[ref3] Baker T. Bester P. A. Sebolai O. M. Albertyn J. Pohl C. H. (2025). Culture-dependent and -independent wastewater surveillance for multiple pathogenic yeasts. J. Fungi 11:86. doi: 10.3390/jof11020086, PMID: 39997380 PMC11856701

[ref4] Bottery M. J. Denning D. W. (2024). Body heat drives antifungal resistance. Nat. Microbiol. 9, 1638–1639. doi: 10.1038/s41564-024-01738-2, PMID: 38961269

[ref5] Cao C. Bing J. Liao G. Nobile C. J. Huang G. (2023). Candida haemulonii species complex: emerging fungal pathogens of the Metschnikowiaceae clade. Zoonoses 3:43. doi: 10.15212/ZOONOSES-2023-0021, PMID: 39238892 PMC11376483

[ref6] Casadevall A. (2018). Fungal diseases in the 21st century: the near and far horizons. Pathog. Immun. 3, 183–196. doi: 10.20411/pai.v3i2.249 30465032 PMC6241320

[ref7] Casadevall A. (2023). Global warming could drive the emergence of new fungal pathogens. Nat. Microbiol. 8, 2217–2219. doi: 10.1038/s41564-023-01512-w, PMID: 38030896

[ref8] Casadevall A. Kontoyiannis D. P. Robert V. (2019). On the emergence of candida auris: climate change, azoles, swamps, and birds. MBio 10, e01397–e01319. doi: 10.1128/mBio.01397-19 31337723 PMC6650554

[ref9] Chen X. F.. (2022). First two fungemia cases caused by Candida haemulonii var. vulnera in China with emerged antifungal resistance. Front. Microbiol. 13:1036351. doi: 10.3389/fmicb.2022.1036351 36466633 PMC9710277

[ref10] Day A. M. McNiff M. M. da Silva Dantas A. Gow N. A. R. Quinn J. (2018). Hog1 regulates stress tolerance and virulence in the emerging fungal pathogen Candida auris. mSphere 3, e00506–e00518. doi: 10.1128/mSphere.00506-18, PMID: 30355673 PMC6200985

[ref11] De Jong A. W.. (2023). Candida khanbhai sp. nov., a new clinically relevant yeast within the Candida haemulonii species complex. Med. Mycol. 61:myad009. doi: 10.1093/mmy/myad009 36694950 PMC9936790

[ref12] Desnos-Ollivier M. Robert V. Raoux-Barbot D. Groenewald M. Dromer F. (2012). Antifungal susceptibility profiles of 1698 yeast reference strains revealing potential emerging human pathogens. PLoS One 7:e32278. doi: 10.1371/journal.pone.0032278, PMID: 22396754 PMC3291558

[ref13] Edlind T. D. Katiyar S. K. (2010). Mutational analysis of flucytosine resistance in Candida glabrata. Antimicrob. Agents Chemother. 54, 4733–4738. doi: 10.1128/AAC.00605-10, PMID: 20823283 PMC2976130

[ref14] Fisher M. C. Alastruey-Izquierdo A. Berman J. Bicanic T. Bignell E. M. Bowyer P.. (2022). Tackling the emerging threat of antifungal resistance to human health. Nat. Rev. Microbiol. 20, 557–571. doi: 10.1038/s41579-022-00720-1, PMID: 35352028 PMC8962932

[ref15] Gade L. Muñoz J. F. Sheth M. Wagner D. Berkow E. L. Forsberg K.. (2020). Understanding the emergence of multidrug-resistant Candida: using whole-genome sequencing to describe the population structure of Candida haemulonii species complex. Front. Genet. 11:554. doi: 10.3389/fgene.2020.00554, PMID: 32587603 PMC7298116

[ref16] Gong Y. Li T. Yu C. Sun S. (2017). *Candida albicans* heat shock proteins and Hsps-associated signaling pathways as potential antifungal targets. Front. Cell. Infect. Microbiol. 7:520. doi: 10.3389/fcimb.2017.00520, PMID: 29312897 PMC5742142

[ref17] Gusa A.. (2023). Genome-wide analysis of heat stress-stimulated transposon mobility in the human fungal pathogen Cryptococcus deneoformans. Proc. Natl. Acad. Sci. U.S.A 120:e2209831120. doi: 10.1073/pnas.2209831120 36669112 PMC9942834

[ref18] Hagiwara D.. (2017). A novel Zn2-Cys6 transcription factor AtrR plays a key role in an azole resistance mechanism of *Aspergillus fumigatus* by co-regulating cyp51A and cdr1B expressions. PLoS Pathog. 13:e1006096. doi: 10.1371/journal.ppat.1006096 28052140 PMC5215518

[ref19] Heilmann C. J.. (2013). Surface stress induces a conserved cell wall stress response in the pathogenic fungus *Candida albicans*. Eukaryot. Cell 12, 254–264. doi: 10.1128/EC.00278-12 23243062 PMC3571293

[ref20] Huang J. Hu P. Ye L. Shen Z. Chen X. Liu F.. (2024). Pan-drug resistance and hypervirulence in a human fungal pathogen are enabled by mutagenesis induced by mammalian body temperature. Nat. Microbiol. 9, 1686–1699. doi: 10.1038/s41564-024-01720-y, PMID: 38898217

[ref21] Ibe C. Munro C. A. (2021). Fungal cell wall proteins and signaling pathways form a cytoprotective network to combat stresses. J. Fungi 7:739. doi: 10.3390/jof7090739, PMID: 34575777 PMC8466366

[ref22] Jacobs S. E. Jacobs J. L. Dennis E. K. Taimur S. Rana M. Patel D.. (2022). Candida auris Pan-drug-resistant to four classes of antifungal agents. Antimicrob. Agents Chemother. 66:e0005322. doi: 10.1128/aac.00053-22 35770999 PMC9295560

[ref23] Kim M. N. Shin J. H. Sung H. Lee K. Kim E. C. Ryoo N.. (2009). Candida haemulonii and closely related species at 5 university hospitals in Korea: identification, antifungal susceptibility, and clinical features. Clin. Infect. Dis. 48, e57–e61. doi: 10.1086/597108, PMID: 19193113

[ref24] Liao H. Lyon C. J. Ying B. Hu T. (2024). Climate change, its impact on emerging infectious diseases and new technologies to combat the challenge. Emerging Microbes Infect. 13:2356143. doi: 10.1080/22221751.2024.2356143 PMC11138229 38767202

[ref25] Lockhart S. R. Etienne K. A. Vallabhaneni S. Farooqi J. Chowdhary A. Govender N. P.. (2017). Simultaneous emergence of multidrug-resistant candida auris on 3 continents confirmed by whole-genome sequencing and epidemiological analyses. Clin. Infect. Dis. 64, 134–140. doi: 10.1093/cid/ciw691, PMID: 27988485 PMC5215215

[ref26] Money N. P. (2024). Fungal thermotolerance revisited and why climate change is unlikely to be supercharging pathogenic fungi (yet). Fungal Biol. 128, 1638–1641. doi: 10.1016/j.funbio.2024.01.005, PMID: 38341269

[ref27] Mora C.. (2022). Over half of known human pathogenic diseases can be aggravated by climate change. Nat. Clim. Chang. 12, 869–875. doi: 10.1038/s41558-022-01426-1 35968032 PMC9362357

[ref28] Muro M. D. Motta F. D. A. Burger M. Melo A. S. D. A. Dalla-Costa L. M. (2012). Echinocandin resistance in two Candida haemulonii isolates from pediatric patients. J. Clin. Microbiol. 50, 3783–3785. doi: 10.1128/JCM.01136-12. 22895037 PMC3486200

[ref29] Paul S.. (2019). AtrR is an essential determinant of azole resistance in *Aspergillus fumigatus*. MBio 10, e02563–e02518. doi: 10.1128/mBio.02563-18 30862750 PMC6414702

[ref30] Robbins N. Caplan T. Cowen L. E. (2017). Molecular evolution of antifungal drug resistance. Ann. Rev. Microbiol. 71, 753–775. doi: 10.1146/annurev-micro-030117-020345, PMID: 28886681

[ref31] Robert V. Cardinali G. Casadevall A. (2015). Distribution and impact of yeast thermal tolerance permissive for mammalian infection. BMC Biol. 13:18. doi: 10.1186/s12915-015-0127-3, PMID: 25762372 PMC4381509

[ref32] Rodrigues L. S. Gazara R. K. Passarelli-Araujo H. Valengo A. E. Pontes P. V. M. Nunes-da-Fonseca R.. (2020). First genome sequences of two multidrug-resistant Candida haemulonii var. vulnera isolates from pediatric patients with Candidemia. Front. Microbiol. 11:1535. doi: 10.3389/fmicb.2020.01535, PMID: 32719671 PMC7350289

[ref33] Schwiesow M. J. W. Elde N. C. Hilbert Z. A. (2024). Distinct routes to thermotolerance in the fungal pathogen Cryptococcus neoformans. *bioRxiv*. Available online at: 10.1101/2024.04.08.588590. [Epub ahead of preprint] PMC12606428 40839773

[ref34] Seagle E. E. Williams S. L. Chiller T. M. (2021). Recent trends in the epidemiology of fungal infections. Infect. Dis. Clin. N. Am. 35, 237–260. doi: 10.1016/j.idc.2021.03.001, PMID: 34016277 PMC10989278

[ref35] Seidel D. Wurster S. Jenks J. D. Sati H. Gangneux J. P. Egger M.. (2024). Impact of climate change and natural disasters on fungal infections. Lancet Microbe 5, e594–e605. doi: 10.1016/S2666-5247(24)00039-9, PMID: 38518791

[ref36] Shields R. K. Nguyen M. H. Press E. G. Kwa A. L. Cheng S. du C.. (2012). The presence of an FKS mutation rather than MIC is an independent risk factor for failure of echinocandin therapy among patients with invasive candidiasis due to Candida glabrata. Antimicrob. Agents Chemother. 56, 4862–4869. doi: 10.1128/AAC.00027-12, PMID: 22751546 PMC3421882

[ref37] Takoutsing B. D. Ooi S. Z. Y. Egu C. B. Gillespie C. S. Bandyopadhyay S. Dada O. E.. (2023). Management and outcomes of intracranial fungal infections in children and adults in Africa: a scoping review protocol. BMJ Open 13:e065943. doi: 10.1136/bmjopen-2022-065943, PMID: 36731932 PMC9896247

[ref38] Vincent B. M. Lancaster A. K. Scherz-Shouval R. Whitesell L. Lindquist S. (2013). Fitness trade-offs restrict the evolution of resistance to amphotericin B. PLoS Biol. 11:e1001692. doi: 10.1371/journal.pbio.1001692 24204207 PMC3812114

